# A high-throughput screening assay to identify inhibitory antibodies targeting alphavirus release

**DOI:** 10.1186/s12985-022-01906-y

**Published:** 2022-10-29

**Authors:** Anushka Ramjag, Sergej Cutrone, Kai Lu, Christine Crasto, Jing Jin, Sonia Bakkour, Christine V. F. Carrington, Graham Simmons

**Affiliations:** 1 Department of Preclinical Sciences, The University of the West Indies, St. Augustine Campus, St. Augustine, Trinidad and Tobago; 2 Vitalant Research Institute, 270 Masonic Avenue, San Francisco, CA 94118 USA; 3 University of California San Francisco, San Francisco, CA 94143 USA

**Keywords:** chikungunya virus, Mayaro virus, Neutralizing antibodies, Anti-budding, HTS assay

## Abstract

**Background:**

Several studies have demonstrated neutralizing antibodies to be highly effective against alphavirus infection in animal models, both prophylactically and remedially. In most studies, neutralizing antibodies have been evaluated for their ability to block viral entry in vitro but recent evidence suggests that antibody inhibition through other mechanisms, including viral budding/release, significantly contributes to viral control in vivo for a number of alphaviruses*.*

**Results:**

We describe a BSL-2, cell-based, high-throughput screening system that specifically screens for inhibitors of alphavirus egress using chikungunya virus (CHIKV) and Mayaro virus (MAYV) novel replication competent nano-luciferase (nLuc) reporter viruses. Screening of both polyclonal sera and memory B-cell clones from CHIKV immune individuals using the optimized assay detected several antibodies that display potent anti-budding activity.

**Conclusions:**

We describe an “anti-budding assay” to specifically screen for inhibitors of viral egress using novel CHIKV and MAYV nLuc reporter viruses. This BSL-2 safe, high-throughput system can be utilized to explore neutralizing “anti-budding” antibodies to yield potent candidates for CHIKV and MAYV therapeutics and prophylaxis.

## Background

The mosquito-borne chikungunya virus (CHIKV) is the etiological agent of chikungunya fever (CHIKF), a febrile illness that is typically accompanied by debilitating myalgia and arthralgia. Although CHIKF is a mostly self-limiting disease, symptoms can be severely incapacitating and persistent. Approximately 40% of infected individuals will experience chronic rheumatic sequelae for years after contracting CHIKV [1]. In some cases, acute infections can be accompanied by more severe symptoms including neurological complications like Guillain-Barré syndrome, cardiovascular difficulties, and death. Rapid spread during epidemics makes CHIKV a particularly formidable virus; studies have reported seroconversion rates ranging from 25% to as much as 90% [2–6]. Cumulative medical costs, decreased productivity, and taxed healthcare services contribute to a significant disease burden. Therefore, despite its low mortality rate, CHIKV is able to exact a substantial socioeconomic impact.

Mayaro virus (MAYV) is the most recent in an unprecedented number of emerging arboviruses to hit the Western hemisphere. The eponymous virus was first discovered in the Mayaro county of Trinidad in 1954 [7] but has since been isolated in South America [8–12] and more recently Haiti [13]. Closely related to CHIKV, MAYV causes similar symptoms, including debilitating arthralgia that can persist for years after infection [12]. The two alphaviral diseases are indistinguishable at presentation, complicating diagnosis particularly in areas of co-circulation with other symptomatically similar arboviruses like dengue virus (DENV) and Zika virus (ZIKV). Although MAYV is presumed to be transmitted by *Hemagogous* mosquitoes to non-human primates, studies have shown the *Anopheline* genus to be competent vectors while *Aedes* mosquitoes (vector to DENV, ZIKV, and CHIKV) can experimentally transmit MAYV [14–16]. Single point mutations can lead to more efficient vector transmission, as with CHIKV and Venezuelan equine encephalitis [17, 18]. A recent retrospective study [19] on sera samples collected during the 2014 CHIKV outbreak in Trinidad and Tobago yielded several MAYV RT-PCR positives, half of which were collected from distinctly urban areas. These data and recent outbreaks highlight the importance of MAYV as an emerging arbovirus with the potential to adapt to other vectors and an urban transmission cycle, facilitating its global spread.

Like other alphaviruses, CHIKV and MAYV are lipid-enveloped positive-sense RNA viruses. Virions enter host cells via receptor-mediated endocytosis using receptor-binding domains in domains A and B of the E2 glycoprotein [20]. Several cellular receptors have been identified [21, 22] including a pan-arthritogenic alphaviral receptor Mxra8 [23] for CHIKV, MAYV, Ross River virus, and O’nyong nyong virus. Once internalized into the host cell, alphaviruses are trafficked to endosomes where the acidic pH triggers conformational changes in the acid-sensitive region (ASR) of the E1–E2 heterodimer. This disassociation exposes a hydrophobic fusion loop in domain II of the E1 protein, allowing insertion into the endosomal membrane. E1 then refolds to form a post-fusion trimeric hairpin-like structure, inducing fusion of viral and host endosome membranes. The viral nucleocapsid is released into the cytosol where viral replication can begin. The alphaviral genome encodes four non-structural and five structural proteins. The latter are translated as a single polypeptide, containing capsid (C) and envelope proteins p62(E3-E2)-6K-E1, which is subsequently proteolytically processed in the endoplasmic reticulum. In particular, the precursor p62 and E1 form heterodimers that further trimerize to form distinctive viral “spikes”, giving rise to a complex icosahedral, glycoprotein shell surrounding the viral membrane and nucleocapsid in the mature virion. The precursor p62 is later cleaved by host furin-like proteases to yield E2 and E3 during trafficking in the Golgi network [24]. The E1 and E2 proteins are often targets of antibody responses, particularly the ASR and exposed viral spikes.

There are currently neither commercially available vaccines nor therapeutic agents for CHIKV or MAYV. Instead, patients are treated symptomatically with acetaminophen or non-steroidal anti-inflammatory drugs (NSAIDs) to manage pain and inflammation. However, neutralizing antibodies (NAbs) against CHIKV, MAYV, and other alphaviruses have been isolated and shown to display potent inhibitory effects, both therapeutically and prophylactically, against infection in animal models [25–34]. Antiviral antibodies are most often screened for their ability to block viral entry (whether via attachment and internalization, or later fusion within acidified endosomes) but there is often little correlation between the potency of in vitro entry neutralization and the ability to prophylactically and/or therapeutically protect in vivo. It is likely that while in vitro entry neutralization may be a useful surrogate of function against native epitopes [35], other mechanisms of protection probably play equal or greater roles in in vivo potency. These generally involve host cell Fc effector functions and include complement- and cell-mediated antiviral mechanisms such as antibody-dependent cellular cytotoxicity (ADCC) and antibody-dependent cellular phagocytosis (ADCP) [36]. Recently, two pan-protective yet poorly neutralizing human mAbs that avidly bind to viral antigen on the surface of cells infected with arthritogenic (CHIKV and MAYV) and encephalitic alphaviruses (Venezuelan, Eastern, and Western equine encephalitis) displayed protective effects in a mouse model through multiple mechanisms, including monocyte-dependent Fc effector functions and inhibition of viral egress [37]. Similarly, another study on E1-specific human mAbs isolated from the B-cells of individuals exposed to Eastern equine encephalitis virus also demonstrated broad protection associated with antibodies that block viral egress but did not require Fc-mediated functions [38]. In fact, several monoclonal antibodies (mAbs) have been shown to more potently inhibit viral release from infected cells compared to entry neutralization [36]. Our group and collaborators previously demonstrated a dual block by CHIKV NAbs C9 [28] and IM-CKV063 on both viral entry and release. Release of virions (as measured by both viral RNA and infectious virus titres in supernatant) by NAb treated cells is dramatically reduced compared to untreated cells for a range of viral strains and NAbs [26, 31]. The NAbs functioned to inhibit viral release by cross-linking and coalescing viral glycoprotein at the cell surface, thus preventing glycoprotein-driven particle formation and budding, resulting in assembled capsid cores remaining arrested beneath the plasma membrane. Furthermore, the coalescence of glycoprotein into large patches by NAbs leads to cross-linking of Fc receptors on immune effector cells and hence highly potent induction of ADCC [30, 36].

The clinical potential of NAbs as prophylactics and therapeutics, and their usefulness in identifying viral epitopes to be targeted in vaccine development and characterization is well accepted. Exploring NAbs that specifically inhibit viral budding is an innovative approach for antibody therapeutics and prophylaxis and can potentially yield even more potent candidates for use in CHIKV protection and/or treatment. Furthermore, identification of NAbs that strongly inhibit budding by creating antibody/antigen complexes on the cell surface will also identify mAbs that will act potently through ADCC in vivo. Here, we describe the development of a BSL-2 safe, cell-based, high-throughput screening (HTS) system for inhibitors of alphavirus egress using novel CHIKV and MAYV nano-luciferase reporter viruses, as well as the use of the resulting “anti-budding assay” to screen both polyclonal sera and memory B-cells from CHIKV recovered individuals.

## Results

### Generation of nano-luciferase (nLuc) reporter viruses

**Fig. 1:**
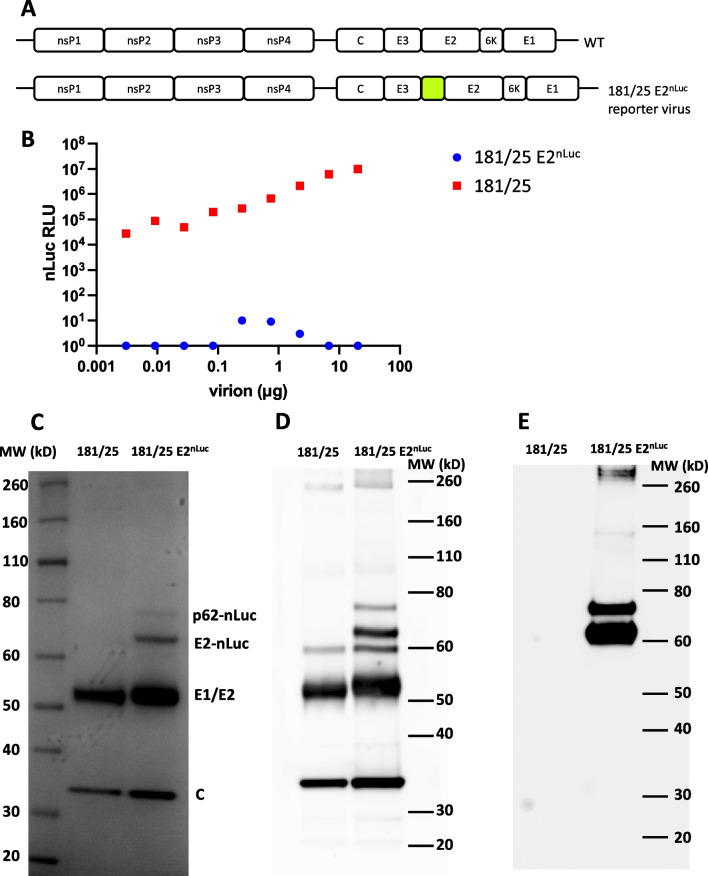
181/25 E2^nLuc^ virus particles carry nano-luciferase activity. **A** Illustration of CHIKV wildtype and nLuc reporter virus genomes. DNA encoding the nLuc reporter (highlighted) was inserted into viral cDNA clones immediately downstream of the furin cleavage site between E3 and E2. **B** Gradient-purified 181/25 and 181/25 E2^nLuc^ viral particles underwent three-fold serial dilutions and nLuc activity was measured. Gradient-purified particles were also separated on a SDS-PAGE followed by **C** Coomassie staining and immuno-blot with **D** anti-CHIKV and **E** anti-nLuc

**Fig. 2:**
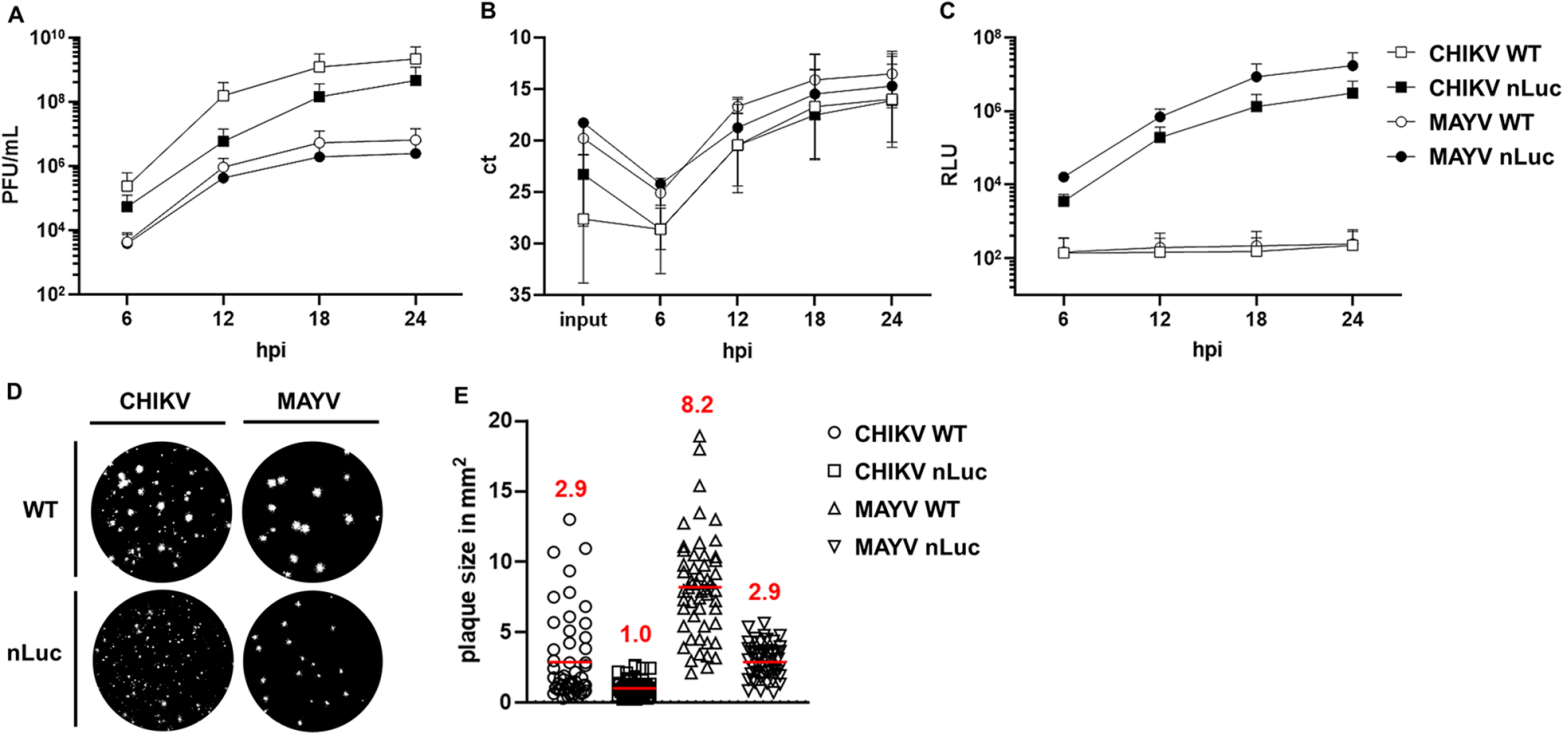
Generation of CHIKV 181/25 E2^nLuc^ and MAYV E2^nLuc^ reporter viruses. BHK-21 cells were infected with CHIKV/MAYV wild-type (WT) or nLuc reporter virus. Supernatant was harvested 6, 12, 18, and 24 hpi (hours post infection) and used to determine **A** infectious titres via plaque forming unit assay in U2OS cells, **B** relative viral RNA levels via qRT-PCR and **C** nLuc activity (represented by RLU) associated with released viral particles. Graphs represent the arithmetic mean ± SD of three independent experiments. Plaque size was compared for WT and nLuc CHIKV/MAYV with examples of plaques (**D**) and by quantitating 53 plaques from each virus as described in the methods (**E**)

We have previously described CHIKV constructs with mCherry fluorescent protein inserted immediately downstream of the furin cleavage site between E3 and E2. This results in functional envelope with the reporter protein fused to the N-terminus of E2 protein [39]. However, there is some attenuation, and if mCherry is used in combination with an already attenuated vaccine strain like CHIKV 181/25, there is a lack of replication [39]. Therefore, to make a BSL-2 safe reporter virus based on CHIKV 181/25, we utilized the smaller reporter protein, nano-luciferase (nLuc). The latter was also mutated to modify a C-terminal cysteine (amino acid 164) to a serine in order to reduce potential adverse effects of a free cysteine on E2 structure without harming catalytic activity [40]. Thus, the nLuc gene was inserted into a cDNA clone of CHIKV vaccine strain 181/25, so that the nLuc enzyme is expressed on the N-terminus of the E2 protein in the resulting reporter virus (Fig. 1A). Similarly, nLuc was positioned in an analogous position in MAYV. Infectious virus could be recovered from both clones, with very high levels of nLuc activity in supernatants (Fig. 2C), suggesting efficient expression and release of particles from infected cells. Measurement of nLuc activity associated with released virus in serial dilutions of supernatant showed that signal was detectable (approximately 250-fold above background) with as little as 7.5 PFU (25824 RLU [relative light units] versus 91 RLU for no virus control). Furthermore, after purification of viral particles, extensive nLuc activity was seen associated with viral particles (Fig. 1B). In keeping with previous findings using mCherry [39], the majority of nLuc associated with mature viral particles was as nLuc-E2 fusion protein, rather than as part of uncleaved p62-nLuc (Fig. 1C–E).

Figure 2A, B show the kinetics of the recovery of replication competent reporter virus from BHK cells in comparison to wild-type virus as measured by PFU/mL and relative viral RNA levels. The similarity in growth curves for each wild-type virus and its reporter virus counterpart indicates that for both MAYV and CHIKV, viral replication is not grossly affected by addition of nLuc at the N-terminus of E2. However, it was noted that plaque size for both CHIKV 181/25 E2^nLuc^ and MAYV E2^nLuc^ were smaller compared to the parental viruses, suggesting some degree of attenuation (Fig. 2D, E).

### Anti-budding assay optimization and validation

**Fig. 3:**
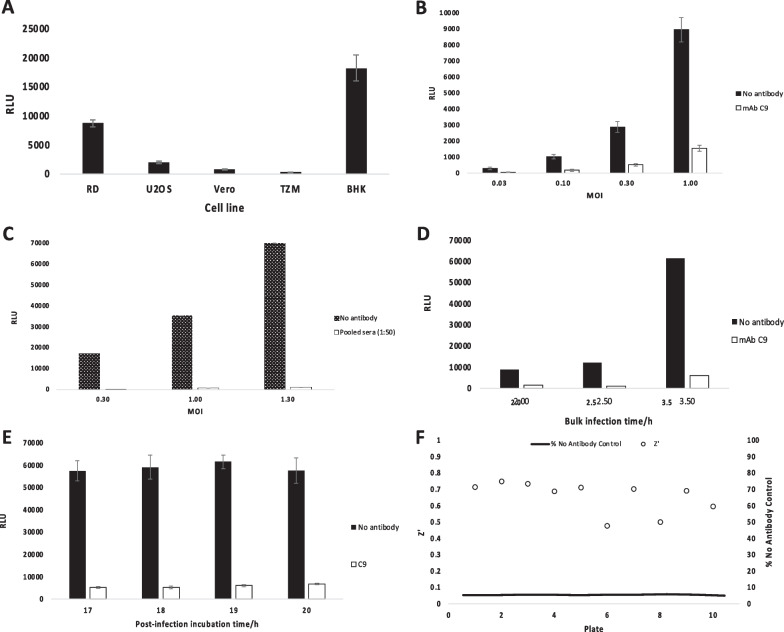
Optimizing parameters for CHIKV and MAYV anti-budding assays. Cells were bulk infected for 3.5 h with CHIKV or MAYV nLuc reporter virus, excess virus washed off, cells plated and incubated for 18 h with or without the addition of control inhibitor, and nLuc activity measured in 50 µL supernatant. **A** Different cell lines were tested for susceptibility to infection by CHIKV nLuc. Cells were infected in bulk and then removed from the plastic surface and counted. Thus, MOIs were calculated retrospectively and ranged from 0.2 (TZM) to 1.3 (U2OS). RD cells were used in subsequent assays to assess MOI ranges for CHIKV (**B**) and MAYV (**C**) reporter viruses using control inhibitors mAb C9 (0.5 µg/mL) or pooled sera (diluted 1:50) respectively. **D** RD cells were incubated with CHIKV nLuc virus for 2–3.5 h at 30 min increments, after which the assay was continued as described. **E** Post bulk infection with CHIKV nLuc, excess virus was washed off and 50 µL supernatant was removed from plated cells at 1 h intervals from 17 to 20 h to measure nLuc activity. **F** Pilot screen demonstrating suitability for HTS screening. Results are shown as percentage of nano-luciferase reading of wells containing no antibody. Infected cells with and without C9 on 10 test plates were subjected to Z′ analysis to determine variation and suitability for HTS systems. A Z′ factor of 0.5–1.0 is deemed sufficiently robust for HTS assays. All results shown carry a CV score of ≤ 15%

To build an assay to screen for anti-viral activity against alphavirus particle release, we infected cells and then plated them into 96-wells followed by addition of the lysosomal agent ammonium chloride (NH_4_Cl) to prevent further rounds of infection. Alphaviral entry is pH-dependent and hence inhibited by NH_4_Cl [41], while viral budding is not [31]. NH_4_Cl was added together with test antibody or sera and incubated until supernatant was removed and interrogated for nLuc activity- and hence the presence of budded virus. Various parameters were tested to determine optimal conditions for this anti-budding assay. Firstly, the suitability of five permissive cell lines (RD, BHK, U2OS, Vero, TZM) was assessed (Fig. 3A). Levels of budded virus (as interpreted by nLuc activity) were highest for RD and BHK cells. RD cells (n = 48 wells) displayed the least intra-assay variation (%CV = 7.45; Z′ = 0.78) compared to BHK cells (n = 48 wells; %CV = 12.16; Z′ = 0.63) and were used in subsequent assays. Optimal viral MOI (multiplicity of infection) was then determined by infecting RD cells with CHIKV and MAYV reporter virus at MOIs ranging 0.03–1.3 (Fig. 3B, C). For CHIKV assays, anti-CHIKV mAb C9 (0.5 µg/mL) was included as a control budding inhibitor. For MAYV assays, sera samples from four volunteers (TT038, TT047, TT052, TT054) were pooled and the pool was used at a final dilution of 1:50. These volunteers were seropositive for CHIKV but were able to neutralize MAYV 12A infection on Vero cells by plaque reduction neutralization tests (PRNT) [42]. Since higher MOIs yielded lower intra-assay variation (n = 48 wells) and higher levels of budded virus, MOI 1–1.5 was used in subsequent assays to maximize comparisons with positive control.

**Fig. 4:**
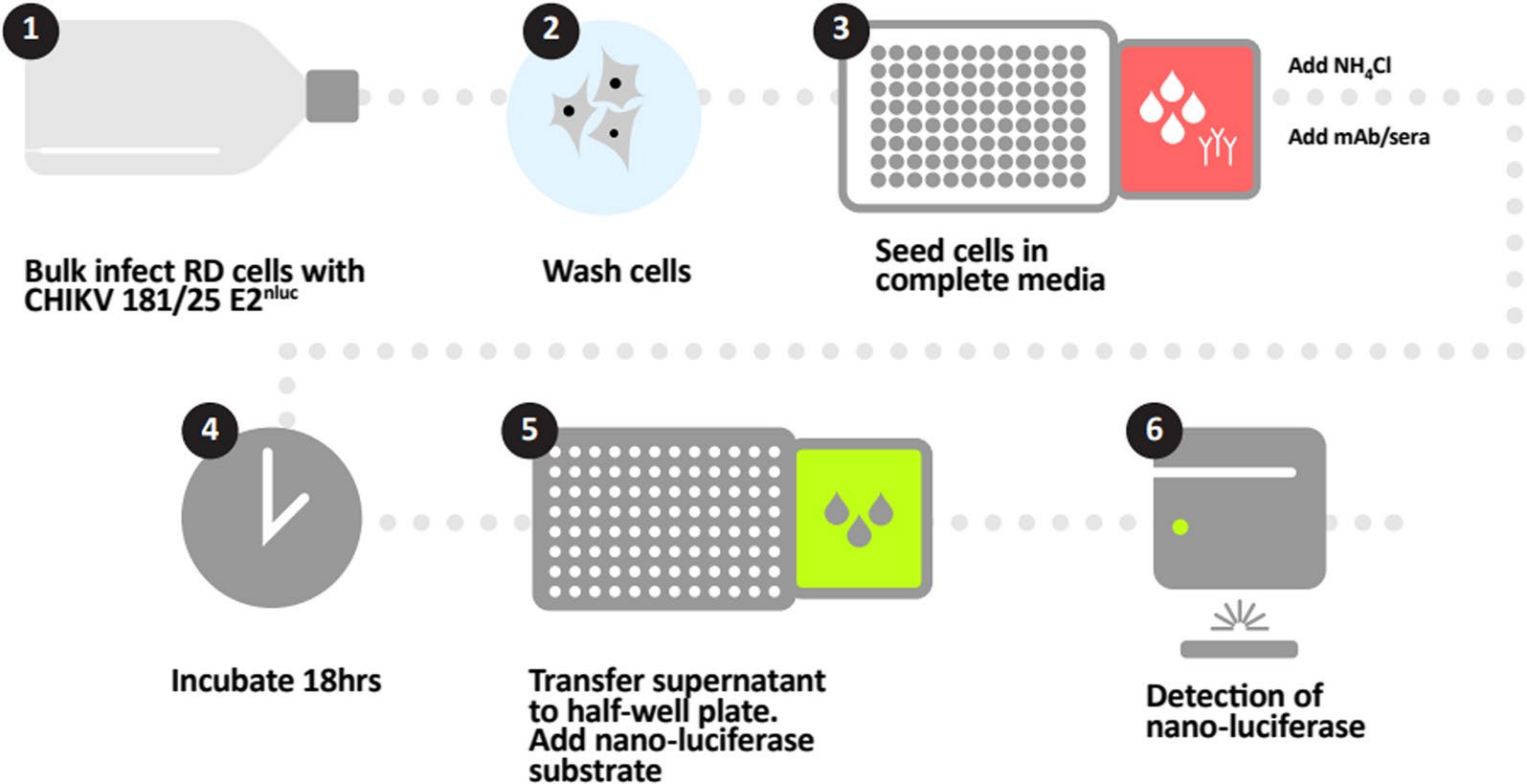
Optimized CHIKV anti-budding assay. The assay uses a novel CHIKV nLuc reporter virus to screen specifically for antibodies that inhibit viral budding. RD cells were first bulk infected with reporter virus, excess virus washed off after 2 h, the monolayer disrupted, and infected cells seeded onto a 96-well plate at 2.5 × 10^4^ cells/well in 100 µL/well of complete growth medium. 100 µL diluted CHIKV-positive human sera or B-cell supernatant was then transferred to assay wells. All wells, including those with positive inhibition control mAb (2 µg/µL C9) and negative control (media only), were supplemented with 20 mM NH_4_Cl. Plates were then incubated for 18 h at 37 °C/5% CO_2_, following which 50 µL of supernatant was harvested from each well and transferred to white half-well plates (Greiner Bio-One) for luminescence readings. For all assays, spectrophotometer gain was adjusted to negative control wells

Next, incubation times for both initial bulk infection of cells with reporter virus (Fig. 4, stage 1) and the later incubation of infected cells and antibody (Fig. 4, stage 4) were assessed. Overall, we observed a sixfold increase in budding between 2 h and 3.5 h incubation (Fig. 3D). We proceeded with a bulk infection time of 2 h for a less time-consuming assay since luminescence values were still reasonably high. In addition, a more favourable level of budding inhibition was observed at 2 h (83%) versus 3.5 h (90%) bulk infection times using C9 as a positive control. Post infection, cells were incubated with and without C9 and luminescence readings were compared at time points from 17 to 20 h (Fig. 3E). There were no significant differences in nLuc activity but since there was evidence of cell death at 19 h and 20 h (cell density was also observably lower with a higher degree of cell detachment), we opted to proceed with 18 h incubation periods where C9 inhibited budding by ~ 91% compared to the control. It was noted that plate type (black or white) did not significantly affect luminescence readings. To demonstrate the effectiveness of this assay as an HTS system, a pilot screen using ten 96-well plates containing assay wells with no antibody were run under the optimized conditions. Sixteen control wells with mAb C9 were included on each plate. Results showed an inter-plate coefficient of variability (CV) of 11.98% and a Z′ of 0.60 (Fig. 3F).

### Screening of polyclonal sera and B-cell supernatants from CHIKV positive individuals

**Fig. 5:**
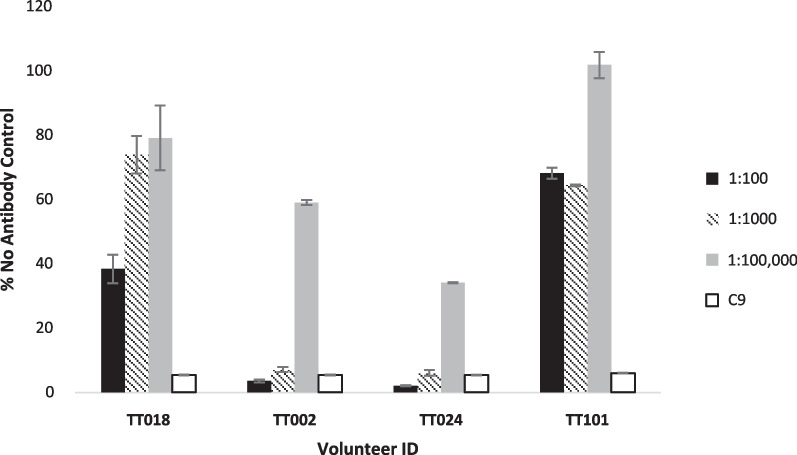
Dose responses for varying dilutions of human polyclonal sera. Samples from three volunteers with previous exposure to CHIKV (TT018, TT002, and TT024) at different times post symptom onset (4, 195, and 240 dpo respectively) were screened using the anti-budding assay system. All volunteers were previously (TT002 and TT024) or at enrollment (TT018) confirmed by RT-PCR. Serum from one volunteer with an ongoing ZIKV infection (5 dpo) and no evidence of any exposure to CHIKV was included as a specificity control (TT101). Different serum dilutions were tested to determine an appropriate dilution range for the anti-budding assay. Positive control mAb C9 (2 mg/mL) was included for comparison. Nano-luciferase readings from control wells containing no antibody were set at 100%. Results were calculated as a percentage of these control wells and represent the mean ± S.D of triplicate measurements. All results shown carry a CV score of ≤ 15%

An appropriate dilution range for screening of polyclonal sera was determined by testing sera collected from three CHIKV-exposed individuals at different days post onset of symptoms (dpo): TT018 (4 dpo), TT002 (195 dpo), and TT024 (240 dpo) respectively (Fig. 5). All three volunteers were previously confirmed as CHIKV positive by RT-PCR prior to enrollment in this study. Serum collected at 5 dpo from a RT-PCR confirmed ZIKV infection in volunteer TT101 (no evidence of exposure to CHIKV) was included as a specificity control.

Sera from the CHIKV-recovered TT002 and TT024 very potently inhibited CHIKV budding, with TT024 able to inhibit budding by greater than 50%, even at a 1:100,000 dilution. In comparison, the acute phase serum (4 dpo) from TT018 was only able to achieve > 50% inhibition at 1:100, while the CHIKV negative control TT101 expectedly did not specifically reduce viral release. For subsequent screens of polyclonal sera, dilutions of 1:100 and 1:1000 were used. The selected concentrations were presumed to minimize the effects of antibody saturation at lower dilutions and allow identification of potent anti-budders which may be masked at higher dilutions.

A total of 47 serum samples from 36 volunteers with suspected or confirmed CHIKV infection were screened using the final anti-budding assay protocol described in Fig. 4. Volunteers were recruited at different times post onset of symptoms (indicative of arboviral infection) and screened for CHIKV using CHIKV-specific IgM and IgG, and RT-PCR if ≤ 30 dpo. Longitudinal samples were collected from eight individuals, including TT095 who had no laboratory evidence of CHIKV exposure at any of the sampled time points, and was included alongside TT101 from above as CHIKV negative controls. Because other arboviruses co-circulate with CHIKV in Trinidad and Tobago (*e.g.* MAYV and ZIKV), samples were considered CHIKV acute only if RT-PCR positive or ≤ 30 dpo with evidence of seroconversion (*i.e.* IgM positive or equivocal) for CHIKV.

Table 1 below shows that 36 samples inhibited CHIKV budding by > 50% (*i.e.* < 50% infection was observed compared to the no antibody control) at a 1:100 dilution with 30 retaining this potency even at dilutions of 1:1000. Five volunteers with longitudinal sampling points (TT002, TT035, TT038, TT047, and TT059) retained significant anti-budding capability (*i.e.* > 70% inhibition) over the time periods represented (10–952 dpo). Two volunteers in acute stages of CHIKV infection (TT015a and TT016a) inhibited virus poorly but potency was greatly improved for longitudinally recovered samples. Contrastingly, three CHIKV-recovered volunteers (TT010, TT068, and TT087) displayed poor to no anti-viral budding capability despite being anti-CHIKV seropositive.

**Table 1:** Polyclonal sera screens using the CHIKV anti-budding assay

Sample ID	DPO	% No antibody control		RT-PCR	IgM	IgG	CHIKV status
		1:100	1:1000				
TT002a	200	3.55	7.09	+ (3 dpo)	−	+	Recovered
TT002b	640	5.46	10.50	+ (3 dpo)	−	+	Recovered
TT003	262	12.45	34.00	+ (4 dpo)	−	+	Recovered
TT009	182	17.94	19.67	+ (7 dpo)	+	+	Recovered
TT010	332	84.79	87.52	− (4 dpo)	±	+	Recovered
TT015a	3	80.93	98.08	−	−	−	Acute
TT015b	141	20.96	32.59	− (3 dpo)	+	+	Recovered
TT016a	3	70.18	88.52	+	+	−	Acute
TT016b	128	16.77	15.36	+ (3 dpo)	±	+	Recovered
TT018	4	38.33	73.85	+	−	−	Acute
TT024	240	2.00	5.97	N/A	−	+	Recovered
TT030	150	32.69	16.73	N/A	−	+	Recovered
TT032	210	3.33	10.14	N/A	+	−	Recovered
TT035a	135	20.77	15.57	N/A	−	+	Recovered
TT035b	302	29.89	23.42	N/A	−	+	Recovered
TT037	210	20.14	10.00	N/A	+	+	Recovered
TT038a	210	26.98	11.10	N/A	−	+	Recovered
TT038b	952	32.98	24.37	N/A	−	+	Recovered
TT039	655	28.70	66.26	N/A	−	+	Recovered
TT046	210	38.78	71.38	N/A	−	+	Recovered
TT047a	45	8.16	11.56	N/A	+	+	Recovered
TT047b	130	5.00	10.00	N/A	+	+	Recovered
TT052	360	6.29	16.27	N/A	±	+	Recovered
TT054	330	2.96	9.98	N/A	±	+	Recovered
TT059a	10	15.11	19.87	−	+	±	Acute
TT059b	145	5.81	15.28	− (10 dpo)	−	+	Recovered
TT059c	284	17.47	12.61	− (10 dpo)	−	+	Recovered
TT065	13	29.41	72.01	−	−	+	Recovered
TT068	212	70.55	62.23	− (5 dpo)	+	+	Recovered
TT070	7	10.65	39.41	−	−	+	Recovered
TT075	12	7.96	36.37	−	−	+	Recovered
TT076	6	6.06	12.28	−	−	+	Recovered
TT078	6	12.80	53.41	−	−	+	Recovered
TT080	11	7.23	20.38	−	±	−	Acute
TT087	150	50.79	103.31	N/A	−	+	Recovered
TT090	460	46.95	42.65	N/A	−	+	Recovered
TT092	360	28.44	49.20	N/A	−	+	Recovered
TT095a	4	92.49	95.52	−	−	−	Negative
TT095b	22	86.41	105.19	−	−	−	Negative
TT095c	147	119.61	125.85	− (4 dpo)	−	−	Negative
TT095d	399	112.67	104.57	− (4 dpo)	−	−	Negative
TT101	5	68.16	64.41	−	−	−	Negative
TT103	30	7.68	16.32	−	−	+	Recovered
TT107	30	8.11	20.72	−	−	+	Recovered
TT114	10	42.24	79.73	−	−	+	Recovered
TT120	11	96.75	101.83	−	±	+	Recovered
TT122	23	35.17	30.08	−	−	+	Recovered

RT-PCR and ELISA results are indicated positive (+), negative (−), or equivocal (±) where applicable. Some volunteers were previously screened by RT-PCR for CHIKV during acute stages of infection. Results from their initial screens are denoted with the dpo at that time. Anti-budding assay results are shown as percentage of nano-luciferase reading of wells containing no antibody (% control) and represent the mean ± SD of triplicate measurements. All results shown carry a CV score of ≤ 15%

**Fig. 6:**
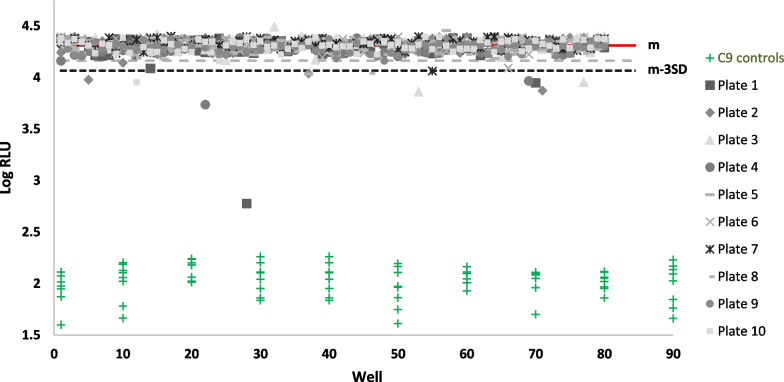
Screening of B-cell supernatants. 800 test wells generated from TT052, an individual one year post onset of CHIKV infection, were screened for anti-budding activity. Results are represented as the logarithm of relative light units (nano-luciferase readings) from ten 96-well plates (inter-plate CV = 14.4%; Z′ = 0.57). Different symbols are used to represent individual plates and associated C9 control wells are shown in green. Lines representing mean readings (red) as well as those 2–3 SD lower than the mean are shown (dotted grey and black respectively). A reading of at least 3SD below the mean was considered a “hit”

Finally, we screened 800 memory B-cell supernatants isolated from PBMCs collected from a 52-year-old CHIKV IgG positive volunteer (TT052) whose serum samples displayed very high anti-budding activity (> 84% inhibition) at one year post onset of illness (Fig. 6). Thirteen [13] clones producing antibodies that exhibited budding inhibition greater than 3 S.D. below the mean were identified. Inhibition and presence of anti-CHIKV IgG was confirmed after expansion of these clones. Cloning of individual mAbs is ongoing.

## Discussion

Identification of novel, potent NAbs for therapeutics and prophylaxis against CHIKV and MAYV has the potential to reduce both the public health and economic impact of these debilitating viruses. In the current study, we developed a novel high-throughput assay system to screen for CHIKV- and MAYV- specific anti-budding antibodies. The system is based on novel reporter viruses constructed by inserting nLuc between E3 and E2 of CHIKV vaccine strain 181/25 (CHIKV 181/25 E2^nLuc^) and MAYV. Results indicate that the dynamics of viral replication were not grossly affected by the addition of nLuc, as both the CHIKV and MAYV reporter viruses demonstrated similar growth kinetics to wild-types. The optimized assays were able to detect nLuc activity above background with as little as 7.5 PFU of reporter virus in the case of CHIKV 181/25 E2^nLuc^. The reporter viruses are fully replication competent; although reduced plaque size suggested some attenuation, possibly in cell–cell spread. The use of the vaccine strain CHIKV 181/25, as well as MAYV, allows the convenience of handling virus at BSL-2. The incorporation of nLuc means that viral budding can be monitored by direct measurement of luminescence in tissue culture supernatant, which is more convenient and suited for efficient generation of results than RT-PCR or plaque assay. The assay employs NH_4_Cl to prevent entry of budded virions (and therefore further rounds of infection). In our studies, NH_4_Cl did not affect viral release to a significant degree [30, 31, 39], however there are some reports of NH_4_Cl suppressing viral secretion from host cells [43]. Thus, the use of other lysosomotropic agents may further enhance this assay. nLuc was selected for its small size (20 kDa), however it is still possible that some unique E2 epitopes are obscured as a result of the fusion. The anti-budding assay system was first used to perform preliminary screens of polyclonal sera from volunteers exposed to CHIKV. Highly potent anti-budding activity was observed in most of the samples screened. Thirty [30] samples from 22 CHIKV positive volunteers were able to reduce infection to ≤ 20% of the control antibody, even at higher dilutions and long after infection *e.g.* up to 952 dpo in volunteer TT038. Two serum samples from volunteer TT002 both showed > 95% budding inhibition despite having been collected over one year apart (200 dpo vs 640 dpo). These results suggest that inhibition of viral budding/release is a major mechanism for viral control, but further work to test in isolation the different mechanisms of anti-viral activity in vivo is necessary to confirm.

Seven sera samples from CHIKV positive volunteers showed poor to no budding inhibition compared to the control, even at higher concentrations. These included two acute samples, TT015a and TT016a, whose accompanying longitudinal recovered samples proved to be highly potent. Two others (TT114 and TT120) were also collected during acute stages (10–11 dpo) but were IgG positive. The three remaining volunteers (TT010, TT068, and TT087) were well into their CHIKV recovery period but lacked significant anti-budding activity. It is possible overall weak or waning responses to CHIKV are responsible for this.

Most of the anti-alphaviral NAbs cited here are IgG which often recognize epitopes on the viral E2 glycoprotein [36]. While most of the sera samples screened with the anti-budding assay were IgG positive for CHIKV, several were concomitantly IgM positive or equivocal, even a full year after symptom onset, as for TT052 discussed below. While the IgM humoral response has been less studied, IgM responses have been shown to provide potent and important protection against other viruses including two other alphaviruses, Sindbis and Semliki Forest virus [44, 45]. The pentamic structure of IgM may allow more effective cross-linking of CHIKV envelope, and hence better budding inhibition compared to bivalent IgG.

Using luminescence as a measure of viral infectivity allowed easy adaptation of our assay for high throughput application. An assay performed with ten (96-well) control plates to demonstrate the HTS potential of the system achieved satisfactory CV (10.82%) and Z′ (0.64) scores. Incorporation of automated liquid handling systems could further increase capacity. HTS assays such as ours can be used to screen donors/volunteers for broadly potent and neutralizing serum responses- a process which was critical to identification of broadly neutralizing antibodies to HIV [46] and more recently with ZIKV [47]. We performed a preliminary B-cell screen of 800 test well mAbs derived from the PBMCs of one individual (TT052) whose serum demonstrated potent anti-budding activity (> 80% budding inhibition compared to the no antibody control) one-year post onset CHIKV infection. Thirteen [13] of the 800 clones demonstrated anti-budding activity at least 3SD below the mean. As budding inhibition is a relatively unexplored area of NAb research, it would be useful to fully elucidate the mechanism of action of these antibodies and map them to viral and cellular epitopes. Our work with mAb C9 suggests that important epitopes may exist only on infected cells and not free virions [31].

The anti-budding assay can be applied further and used for screening and characterizing antiviral potency in drug candidate studies or adapted for use in vaccine development trials by monitoring serum neutralizing potential or B-cell responses in vaccinated subjects. Additionally, candidate antiviral/antibody can be added prior to bulk infection with virus to explore entry neutralization capabilities and thus fully characterize candidate NAbs or lead candidates in compound libraries. Promising clones can be subjected to antibody heavy and light chain variable domain sequencing as previously described [28]. Lead candidate mAbs from these in vitro screens can then be advanced to small animal models of CHIKV for more realistic in vivo prophylactic and therapeutic studies. For a more comprehensive evaluation, particularly in the case of drug screens, cytotoxicity data can be generated alongside luminescence readings to examine the effect of assay conditions and test compound on target cells [48]. In the current study, visual estimations showed that cell density remained > 90% at the 18 h end point.

Lastly, it should be noted that while all sera with significant anti-budding capabilities were CHIKV positive by RT-PCR or ELISA, it is possible that antibody responses were due to another alphavirus. Cross-reactive anti-arboviral antibodies, particularly between CHIKV and MAYV, have been well documented [26, 27, 42, 49] and include cross-reactive anti-budding antibodies [37]. MAYV has been found in co-circulation with CHIKV in Trinidad and Tobago [19].

## Conclusions

We describe a BSL-2, cell-based, high-throughput screening system that specifically screens for inhibitors of alphavirus egress. The assay system utilizes novel CHIKV and MAYV nLuc reporter viruses that are fully replication competent. Screening of both polyclonal sera and memory B-cell clones from CHIKV immune individuals using the optimized assay detected several antibodies that display potent anti-budding activity.

## Materials and methods

### Cell culture

For optimization assays, African green monkey kidney cells (Vero; ATCC CRL-1586), golden hamster kidney cells (BHK-21; ATCC CCL-10), human bone cells (U2OS; ATCC CRL-3455), human rhabdosarcoma cells (RD; CCL-136), and a HeLa (human uterine cells)- derived cell line (TZM; ARP-8129) were used. Cell lines were cultured in Dulbecco’s minimal essential medium (DMEM)/High Modified (HyClone) supplemented with 1% 100X GlutaMAX-I (Gibco), and 10% fetal bovine serum (FBS; Peak Serum) and maintained at 37 °C in a fully humidified atmosphere with 5% CO_2_. For bulk infections, media supplemented with 2% FBS was used.

### Reporter virus production

A novel CHIKV nLuc reporter virus (CHIKV 181/25 E2^nLuc^) that is fully replication competent but attenuated in vivo was constructed by inserting nLuc between E3 and E2 of CHIKV vaccine strain 181/25 (GenBank accession no. L37661, listed as TSI-GSD-218; BEI Resources, NIAID, NIH: NR-13222)—an attenuated strain derived from parental AF15561, a wild-type CHIKV isolated from Thailand [50]. Similarly, MAYV E2^nLuc^ was created using the FLIC infectious clone (kindly supplied by the World Reference Center for Emerging Viruses and Arboviruses. Highly related to GenBank accession no. MK070491.1). The resulting reporter plasmid DNAs were linearized by NotI-HF or PacI digestion for CHIKV or MAYV respectively and then transcribed using SP6 mMESSAGE mMACHINE (Invitrogen). RNA was purified with a MEGAclear kit (Invitrogen) and quantified using NanoDrop ND-1000 (Thermo Scientific). For transfection, 20 μg RNA was electroporated (2 × 4 mm cuvettes, 500 μL/cuvette; 250 V, 15 ms, 2 pulses at 0.1 s interval) into 1 × 10^7^ BHK cells resuspended in 1 mL OptiMEM (Gibco). Transfected cells were seeded into complete medium and reporter virus was harvested at 72 h post transfection by filtering cell culture supernatant through a 0.45 μm filter then stored at − 80 °C until use.

Infectious titres were determined by plaque assay. Plates (6-well or 12-well; Costar) containing Vero or U2OS cell monolayers were incubated with serial dilutions of reporter virus for 1 hour at 37 °C. Residual infected media was then aspirated, and cells overlaid with DMEM supplemented with 2% FBS and 0.8% agarose. Cells were maintained at 37 °C in a fully humidified atmosphere with 5% CO_2_. Plaques were counted three days later and plaque forming units per milliliter (PFU/mL) calculated. Virus was purified using two-step ultracentrifugation as previously described [39], and subjected to electrophoresis with 4 ± 12% sodium dodecyl sulfate–polyacrylamide gel (ThermoFisher Scientific) followed by Coomassie staining with SimpleBlue SafeStain (ThermoFisher Scientific) or Western blot analysis with rabbit polyclonal anti-CHIKV 181/25 (IBT Bioservices) and mouse monoclonal anti-nLuc (Bio-Techne).

### Growth curve

BHK-21 cells were seeded into a 24-well plate and allowed to reach a confluency of 80–90%. After determining the cell count, the cells were infected with virus at a MOI of 0.1 in a total volume of 200 µL for 1 h at 37 °C. The inoculum was subsequently removed and replaced with 500 µL of fresh medium. Supernatant was harvested every 6 h post infection (6, 12, 18, and 24 hpi). Harvested supernatants were analyzed by plaquing, RNA viral load and nLuc activity. Plaquing was performed on U2OS cells in 12-well plates as described above, while for nLuc activity 5 µL supernatant was mixed with 45 µL 1X Luciferase cell lysis buffer (Thermo Scientific), before addition of 50 µL of Nano-Glo luciferase substrate (Promega). Luminescence measurements were made using a POLARstar OPTIMA spectrophotometer (BMG Labs).

### Quantitative real-time RT-PCR

For qRT-PCR, viral RNA was extracted using QIAamp Viral RNA Mini Kit (QIAGEN). For each sample, 50 µL was processed. Viral RNA was amplified by one-step real-time RT-PCR using SuperScript® III Platinum® One-Step qRT-PCR Kit (Life Technologies). For each amplification well, 10 µL of viral RNA diluted 1:10 was added to 12.5 µL of 2X reaction mix, 0.25 µL of 100 µM forward primer, 0.25 µL of 100 µM reverse primer, 0.15 µL of 25 µM probe, 0.5 µL of SuperScript® III/ Platinum® Taq Mix, and 1.35 µL of nuclease-free water. Amplification conditions were: 1 cycle of 50 °C for 30 min (RT reaction) and 95 °C for 2 min (RT inactivation), followed by 45 cycles of 95 °C for 15 s and 60 °C for 1 min. Amplification was performed on a Roche LightCycler 480 II instrument. For the CHIKV assay, the following primers and probe, targeting a region of the nsP4 gene between nucleotides 7421 and 7580, were used: CKV_For (5′ ATG GCC ACC TTT GCA AGC TC 3′), CKV_Rev (5′ GGG ATG AAC TCC ATT GTA GC 3′) and CKV_Probe-FAM (5′ AGG TAC GCA CTA CAG CTA CC 3′). For the MAYV assay, the following primers and probe, targeting a region of the E2 gene between nucleotides 8690 and 8817, were used: MAYV-F (5′ GTG GTC GCA CAG TGA ATC TTT C 3’), MAYV-R (5′ CAA ATG TCC ACC AGG CGA AG 3′) and MAYV-Probe-FAM (5′ ATG GTG GTA GGC TAT CCG ACA GGT C 3′).

### Plaque size estimation

1 × 10^6^ U2OS cells were seeded per well of a 6-well plate and cultivated until confluent. A 1:10 dilution series CHIKV and MAYV (WT or nLuc) was performed using complete cell culture medium and 250µL used per well to infect cells for 1 h at 37 °C. The inoculum was removed and 3 mL overlay medium (1 × MEM, % FBS, 0.6% oxoid agar) added. The plates were incubated for 3–4 days and then fixed with 4% PFA for 30 min at room temperature. PFA and agarose overlay was removed and the fixed monolayer stained with 1% crystal violet, rinsed with water and dried. Photographic documentation of the plaques was performed (Evos xl) with a ruler placed next to the wells at the same height as the monolayer. Images were converted into duotone mode (black and white only) via ImageJ and the pixel number of white-colored plaques was subsequently converted into mm^2^ using the ruler as reference.

### Anti-budding assay: optimization and validation

The anti-budding assay involved bulk infection of CHIKV susceptible cells with reporter virus (diluted in DMEM with 2% FBS) at 37 °C/5% CO_2_ followed by trypsinization and resuspension in complete growth medium (with two washes at 1200 rpm for 5 min each) to facilitate transfer of infected cells to a 96-well tissue culture plate (2.5 × 10^4^ cells per well in 100 μL of complete growth medium) and incubation with 100 μL of test sera or mAbs in the presence of NH_4_Cl. Anti-budding activity was then measured by comparing the amount of reporter virus released into the supernatant by cells in the presence and absence of antibody. Virus was quantified by measuring nLuc reporter expression levels in 50 μl of supernatant using the protocol described above. The inhibitory mAb C9, produced as described in [28] was used as a negative control during optimization assays then later as a positive control for comparison with other mAbs and polyclonal sera.

To optimize the assay, several parameters were varied including: (1) cell types (RD, BHK, U2OS, Vero, and TZM cells), (2) reporter virus MOI (dilutions ranging from 0.03 to 1.30), and incubation times for both (3) bulk infection of cells (ranging from 2 to 4 h, in 30 min increments) and (4) test antibody (17–20 h). NH_4_Cl concentration (10 mM, 20 mM) and plate colour (black vs. white) for luminescent readings were also assessed.

As the nLuc reporter is covalently fused to the virus envelope protein, luminescence readings (measured in RLU) directly reflect the efficiency of viral budding. Readings from wells containing no antibody (*i.e.* negative controls) were averaged and set as 100% infection. Wells containing positive control C9, were calculated as a percentage of the no antibody control.

The suitability of this method as a high-throughput assay was determined by calculating Z prime values (Z′). Statistical calculations were performed in excel as follows (where SD is standard deviation): Z′ = 1 − [(3 × (SD of the C9 positive inhibitory control + SD of the no antibody negative control))/|(mean of the C9 positive inhibitory control—mean of the no antibody negative control)|]. Generally, a Z′ value for each plate of 0.5–1 is indicative of an adequate assay (Zhang et.al. 1999). Co-efficient of variation (CV) scores (calculated as a percentage: 100 x [SD/mean]) were used to measure variation between replicate test wells. A CV score of ≤ 15% was considered acceptable.

### Blood collection and isolation of serum, plasma and PBMCs

Whole blood was obtained from individuals ≥ 18 years of age either presenting with a suspected CHIKV infection or with a history of CHIKV infection in Trinidad and Tobago during the period November 2014 to August 2017. Where possible, follow up samples were periodically collected from donors. PBMC isolation was carried out using SepMate (StemCell Technologies) tubes as described by the manufacturer. Serum or plasma samples were screened for IgM and IgG anti-CHIKV antibodies using ELISA kits from EuroImmun as per manufacturer’s instructions. Samples collected ≤ 30 dpo were also screened by qRT-PCR for the presence of CHIKV nucleic acids as previously described by [51].

### Memory B-cell cloning

Memory B-cells in PBMCs from individual TT052 (a 52-year-old, CHIKV IgG positive male, one year post onset of illness at the time of enrollment into the study) were selectively activated following an adapted version of the protocol previously described [28]. Briefly, B-cells were isolated from cryopreserved PBMCs using a human B-cell isolation kit II (Miltenyi Biotech) following manufacturer’s instructions. Isolated B-cells were seeded with irradiated EL-4-B5 feeder cells in medium containing IL-2 and R848 (to selectively drive memory B-cell proliferation and induce IgG secretion), and B95-8 cell supernatant for immortalization.

### Screening of polyclonal sera and B-cell clones for anti-budding antibodies

Dilutions of serum/plasma samples from 36 individuals with or without evidence of CHIKV infection at the time of sampling, and supernatants from 800 B-cell clones from a CHIKV positive volunteer TT052 (collected two weeks after culturing) were screened for CHIKV anti-budding activity using the optimized anti-budding assay. We found no observable differences between sera and plasma samples during optimization of the assay and so plasma was substituted where serum was in low supply. Luminescence readings from wells containing no antibody (*i.e.* negative controls) were averaged and set as 100% infection. Test wells, including those containing positive control C9, were calculated as a percentage of the no antibody control. Sera was considered positive for anti-budding activity if relative infection levels were ≥ 50% lower than no antibody control and monoclonal antibodies from B-cell supernatants were considered positive if readings were 3SDs below the no antibody control mean (mean_negative_-3xSD).

## Data Availability

Data generated or analysed during this study are included in this published article. Any additional dataset required are available from the corresponding author on reasonable request.
